# Development and validation of an automated and high-throughput quadruplex RT–ddPCR assay for the detection of influenza A, influenza B, respiratory syncytial virus, and SARS-CoV-2

**DOI:** 10.3389/fcimb.2025.1529336

**Published:** 2025-03-28

**Authors:** Zhongqiang Huang, Jian Song, Chunli Shi, Xiaoyu Fan, Yanqun Xiao, Xueliang Wang

**Affiliations:** ^1^ Department of Molecular Biology, Shanghai Centre for Clinical Laboratory, Shanghai, China; ^2^ Department of Quality Control Material R&D, Shanghai Center for Clinical Laboratory, Shanghai, China; ^3^ Department of Molecular Diagnostic Innovation Technology, Shanghai Academy of Experimental Medicine, Shanghai, China

**Keywords:** droplet digital PCR, RT-PCR, respiratory viruses, multiplex assay, QX ONE system

## Abstract

The main detection method for viral respiratory infections is reverse transcription polymerase chain reaction (RT–PCR), but it is susceptible to sample inhibitors and relies on a standard curve and subjective thresholds to quantify nucleic acid targets. However, droplet digital PCR (ddPCR), the third generation of PCR with higher sensitivity and accuracy, is an effective tool for the detection and absolute quantification of respiratory viruses. In this study, we introduced AHQR–ddPCR, which is an automated and high-throughput quadruplex reverse transcription ddPCR assay based on the QX ONE platform for the detection of influenza A, influenza B, respiratory syncytial virus, and SARS-CoV-2 in a single reaction. The AHQR–ddPCR assay had analytical sensitivity as low as 0.65–0.78 copies/μL for four respiratory viruses, and exhibited excellent analytical specificity, intraassay and interassay precision, and a wide linear range for each viral target. The results in clinical samples showed that the assay had good concordance and better diagnostic sensitivity compared to RT-PCR. In short, the highly sensitive and absolutely quantitative AHQR–ddPCR assay has excellent analytical and clinical performance, and the advantage of detecting weakly positive samples, which can effectively reduce false-negative results and is a powerful complement to RT–PCR. In addition, it has great value for virology research and the development of automated molecular assays.

## 1 Introduction

Viral respiratory infections (VRIs) are the commonest acute infectious diseases and one of the leading causes of morbidity and mortality worldwide, especially in children and the elderly (Clementi et al., 2021). The symptoms of VRIs, including fever, cough, and fatigue, are often similar and overlapping, which increases the difficulty of identifying the source of infection. Moreover, co-infection with multiple pathogens can lead to more complex conditions and even delay treatment (Swets et al., 2022). Therefore, the accurate, rapid, and reliable identification of viruses is crucial for the early diagnosis and treatment of infected patients, epidemiological surveillance, and controlling viral transmission.

According to the Centers for Disease Control and Prevention (CDC) and the Chinese CDC, influenza virus (IFV) and respiratory syncytial virus (RSV) had the highest positivity rates among all viruses causing respiratory infections before the coronavirus disease (COVID-19) pandemic (Olsen et al., 2021; Li et al., 2021). The commonest types of human IFV are influenza A virus (IFA) and influenza B virus (IFB), which display frequent genetic mutations and cause seasonal outbreaks with nearly 300,000–650,000 deaths every year associated with seasonal influenza globally (Iuliano et al., 2018). RSV is the most common virus causing bronchiolitis and pneumonia in children worldwide, with the highest prevalence and 2% mortality in children under 5 years of age (Li et al., 2022). The global pandemic of severe acute respiratory syndrome coronavirus 2 (SARS-CoV-2) has also taken an extreme toll in recent years, with more than 6.9 million deaths recorded globally since December 2019 (World Health Organization, 2024). In short, the enormous harm caused to global public health by these viruses emphasizes the importance of laboratory diagnostics to identify pathogens, especially during seasonal epidemics and catastrophic pandemics.

The detection methods for VRIs have evolved over the years, from viral culture to direct fluorescent antibody (DFA) tests and rapid antigen tests (RATs), each with its own characteristics (Zhang et al., 2020). Although viral culture is still regarded as the gold standard for the diagnosis of VRIs, it is too time-consuming and complex to be used for routine diagnoses. DFATs and RATs have the advantages of good specificity, rapid turnaround time, and low cost, but their unsatisfactory sensitivity is prone to false-negative results, which limits their clinical application. With the development of molecular diagnostic techniques, real-time reverse transcription polymerase chain reaction (RT–PCR) has been widely used for the diagnosis of VRIs because its throughput, specificity, and sensitivity are higher than those of the traditional methods mentioned above, and it is even used as the gold standard for the diagnosis of SARS-CoV-2 (World Health Organization, 2024). However, RT–PCR has its own limitations: it is easily affected by inhibitors in the samples, less precision for low-concentration samples, and relies on a standard curve and subjective cut-off values to quantify nucleic acid targets (Buchan and Ledeboer, 2014). In contrast, droplet digital PCR (ddPCR) can redresses the shortcomings of RT–PCR with the principles of limiting dilution, end-point PCR, Poisson statistics and oil-water emulsions, which could counteract the effects of poor amplification efficiency and inhibitors. Therefore, it achieves the absolute quantification of low viral loads in complex background with high precision and sensitivity (Zhang et al., 2022).

As an important diagnostic advancement, multiplex PCR enables the detection of multiple pathogens in a single test, thereby identifying co-infections with multiple pathogens, reducing detection time, and saving precious sample. However, ddPCR still faces some challenges in multiplex detection. For example, the widely used QX200 Droplet Digital PCR System™ (Bio-Rad, California, USA) has only two-color fluorescence channels (Tan et al., 2023), which limits its ability for multiplex detection. To achieve higher-order multiplex assays, several studies have used alternative strategies based on probe mixing or amplitude-based multiplexing (Whale et al., 2016), but the complex optimization process has extremely high requirements in terms of operator ability. In contrast, the new-generation QX ONE Droplet Digital PCR System™ (Bio-Rad, California, USA) (BioRad, 2024) has independent four-color fluorescence channels, making the development and application of multiplex assays easier. Furthermore, this system has the advantages of automation and high throughput compared with most other ddPCR platforms, which greatly reduces errors introduced by inexperienced experimenters during complex operations and minimizes the resources required, ultimately paving the way for widespread use of ddPCR.

To date, there have been no reports of the multiplex detection for respiratory viruses using the QX ONE system. Therefore, we attempted to develop a multiplex reverse transcription ddPCR (RT–ddPCR) assay for the simultaneous detection of IFA, IFB, RSV, and SARS-CoV-2 in one reaction. In summary, we reported the development and analytical validation of an automated and high-throughput quadruplex RT‐ddPCR assay (AHQR–ddPCR), evaluated the clinical performance of this assay and compared the results to those obtained by RT–PCR using retrospective clinical samples. The results demonstrated that the assay can be used for absolute quantification of respiratory viruses and provides a more sensitive and reliable detection method for VRIs.

## 2 Materials and methods

### 2.1 Reference materials and inactivated viruses

Commercial reference materials with known concentrations, developed by the Chinese National Institute of Metrology (CNIM), were used for the analytical validation of the AHQR–ddPCR assay. IFA and IFB were inactivated viruses of subtype H1N1 (code NIM-RM4054) and the Victoria lineage (code NIM-RM4056) respectively, with the concentrations of 1.71 × 10^3^ copies/μL and 2.77 × 10^3^ copies/μL respectively. RSV was the RNA transcripts encoding a portion of the A isolate genome (code NIM-RM4057) with a concentration of 6.10 × 10^4^ copies/μL. SARS-CoV-2 was the purified full-length RNA genome (code GBW (E) 091099) with a concentration of 3.30×10^3^ copies/μL. In addition, other common inactivated pathogens purchased from the Shanghai Institute of Immunity and Infection of the Chinese Academy of Sciences (SIII-CAS) were used to assess the analytical specificity, including Parainfluenza virus (PIV), Adenovirus (ADV), Rhinovirus (RhV), Human Cytomegalovirus (HCMV), Epstein-Barr virus (EBV), Human Metapneumovirus (HMPV), Mycoplasma pneumonia (MP), Chlamydophila pneumonia (CP), Staphylococcus aureus, and Streptococcus pneumonia.

### 2.2 RNA extraction

Viral RNA of all samples was extracted and purified with the EX3600 Automatic Nucleic Acid Extraction and Purification System (Shanghai Liferiver Bio-Tech Corp., Shanghai, China), according to the manufacturer’s instructions. The 300 μL samples were used as raw material to obtain 50 μL eluted RNA samples, and the prepared RNA samples were stored at -80°C before further analyses.

### 2.3 AHQR–ddPCR assay

The generic primers and probes of four viruses were designed based on the Chinese CDC and a previously published study (Bashir et al., 2013; National Institute for Viral Disease Control and Prevention, 2020; Chinese National Influenza Center, 2017), including the matrix protein (M) gene region of IFA, the nonstructural protein (NS) gene region of IFB, the matrix protein (M) gene region of RSV, and the open reading frame 1ab (ORF1ab) gene region of SARS-CoV-2, respectively. All primers and probes were synthesized by Integrated DNA Technologies (Iowa, USA) and shown in **Table 1**.

**Table 1:** Primers and probes used in the AutoHQ RT–ddPCR assay.

Target	Primer/probe	Sequence (5’ to 3’)	Probe dye(s)	Concentration (nM)
Influenza A-M	Forward	GACCRATCCTGTCACCTCTGAC	5’-Cy5/TAO/3IAbRQ-3	600
	Reverse	GGGCATTYTGGACAAAKCGTCTACG		600
	Probe 1	TGCAGTCCTCGCTCACTGGGCACG		100
Influenza B-NS	Forward	TCCTCAACTCACTCTTCGAGCG	5’-HEX/ZEN/3IABkFQ-3’	600
	Reverse	CGGTGCTCTTGACCAAATTGG		600
	Probe 2	CCAATTCGAGCAGCTGAAACTGCGGTG		200
RSV-M	Forward	GGCAAATATGGAAACATACGTGAA	5’-Cy5.5/3IAbRQ-3	600
	Reverse	TCTTTTTCTAGGACATTGTAYTGAACAG		600
	Probe 3	CTGTGTATGTGGAGCCTTCGTGAAGCT		200
SARS-CoV-2-ORF1ab	Forward	CCCTGTGGGTTTTACACTTAA	5’-FAM/ZEN/3IABkFQ-3’	600
	Reverse	ACGATTGTGCATCAGCTGA		600
	Probe 4	CCGTCTGCGGTATGTGGAAAGGTTATGG		100

nM, nmol/L; FAM, 6-carboxyfluorescein (FAM); Cy5, Cyanine 5; HEX, Hexachlorofluorescein; Cy5.5, Cyanine 5.5; IABkFQ, Iowa Black FQ quencher; IAbRQ, Iowa Black RQ quencher; ZEN and TAO are quenchers of double-quenched probes between the 9th and 10th nucleotide.

A standard PCR reaction mix for the assay was prepared by the One-Step RT–ddPCR Advanced Kit for Probes (Bio-Rad, California, USA). Each reaction contained the following components: 5 μL of RNA template, 5 μL of supermix, 2 μL of reverse transcriptase (20 U/μL), 1 μL of dithiothreitol (DTT, 15 mM), 4.8 μL of primer mix, 0.8 μL of probe mix, and 1.4 μL of nuclease-free water to a final volume of 20 μL. The thoroughly blended PCR mixture was then transferred into a system-specific GCR96 Cartridge and detected in the QX ONE system. The program of thermal cycling was 25°C for 3 min, 48°C for 20 min, 95°C for 5 min, and 45 cycles of 95°C for 15 s and 55°C for 30 s, followed by 98°C for 10 min and 25°C for 1 min. A temperature ramp of 2°C/s was set to all steps, and the heated lid temperature was set at 105°C. The quantitative detection results were automatically analyzed by QX ONE Software (version 1.1) based on the fluorescent signals and expressed as copies per microliter of the reaction mixture, which was then converted to copies per microliter of sample with the external equation:


$$\begin{array}{c} copies\;per\;\mu L\;sample\\ =\frac{copies\;per\;\mu L\times 20\;\mu L\;mixture}{5\;\mu L\;RNA\;input}\\ \times \frac{50\;\mu L\;RNA\;elution\;volumes}{300\;\mu L\;sample\;volumes} \end{array}$$

### 2.4 Analytical sensitivity and specificity

The limit of blank (LoB) and the limit of detection (LoD) of the developed AHQR–ddPCR assay were estimated based on the Clinical and Laboratory Standards Institute (CLSI) guidelines (Pierson-Perry et al., 2012). LoB was assessed with five blank samples, and each sample was tested in a total of 12 replicates over 3 consecutive days. The data were finally evaluated by the nonparametric data analysis option, and the 95th percentile result was taken as the LoB.

To determine the LoD, the four viral samples were mixed and serially diluted with nuclease-free water to 3.00, 1.50, 0.75, 0.38, and 0.19 copies/μL. Each dilution was tested by the assay in a total of 30 replicates over 5 consecutive days. The LoD was defined as the concentration of the lowest dilution that could be detected with ≥ 95% probability and calculated by Probit analysis (Burd, 2010), which is a type of regression analysis commonly used in molecular assays to empirically determine the lowest concentration of analyte that can be reliably detected, especially for infectious disease agents. The analytical specificity of the assay was also assessed with the reference materials and inactivated viruses described above.

### 2.5 Intraassay and interassay precision

The intraassay precision and interassay precision of the assay were assessed by analyzing two samples of different concentrations prepared from a mixture of the four reference materials (IFA, IFB, RSV, and SARS-CoV-2) and calculating the mean number of copies and the coefficient of variation (CV) according to the CLSI guidelines (Mcenroe et al., 2014). Intraassay and interassay precision were assessed with 20 replicates of each concentration in the same RT–ddPCR run and 50 replicates over 5 consecutive days, respectively.

### 2.6 Linear dynamic range

The linear dynamic range of the assay was evaluated with serial 2-fold dilutions of the pooled reference materials. All reactions were performed in triplicate, and then the linear regression analysis was performed for the four targets and the correlation coefficient was determined (Mcenroe et al., 2020).

### 2.7 Application to clinical samples

A total of 247 de-identified throat swab samples were collected from October 2023 to April 2024, and January 2025. All samples placed in disposable sampling tubes containing 2.0 mL of virus preservation solution, and then stored at -20°C, these samples had been previously tested using PCR methods, including samples infected with IFA, IFB, RSV, SARS-CoV-2, PIV, ADV, HMPV, or MP.

Each sample was tested in parallel with the assay developed here and commercial RT–PCR kits approved by National Medical Products Administration (NMPA), including Sansure kits (Sansure Biotech Inc, Hunan, China) for IFA, IFB, RSV, SARS-CoV-2, ADV, RhV, and MP, and BioGerm kits (BioGerm Medical Technology, Shanghai, China) for PIV and HMPV. According to the manufacturer’s instructions, the LoD of the comparator assay for IFA, IFB, RSV, and SARS-CoV-2 were 500 copies/mL, 500 copies/mL, 500 copies/mL, and 200 copies/mL, respectively, and the overall coincidence rate of each pathogen was about 99.60%. All RT–PCRs were performed on the QuantStudio 5 Real Time PCR System™ (Thermo Fisher Scientific, Massachusetts, USA). Inconsistent results were re-analyzed for confirmation using targeted next generation sequencing (tNGS) with the NextSeq 550 System (Illumina, Shanghai, China) by KingMed Diagnostics Group Co., Ltd (Shanghai, China), and the LoD of tNGS was 100 copies/mL. Fastp (version 0.20.1) was used to trim adapter sequences and remove low-quality sequences, and the remaining sequences were mapped to the reference sequences with Bowtie 2 (version 2.4.1). The reference sequences used for read mapping were obtained from a database curated from multiple sources, including the GenBank, RefSeq, and NT databases of the National Center for Biotechnology Information (Chen et al., 2018).

### 2.8 Data analysis

Basic statistical analyses and graph construction, including mean number of copies, standard deviation (SD), CV, and linear regression were performed with the Excel 2016 (Microsoft, Washington, USA) and Adobe Illustrator software (Adobe, California, USA). The Probit analysis of LoD and an agreement analysis were performed with SPSS Statistics 23.0 (IBM, New York, USA). The diagnostic performance, and the kappa [*K*] index used to compare the overall agreement/concordance of results between RT–ddPCR and RT–PCR assays were calculated by MedCalc. Differences were considered statistically significant at *P*<0.05.

## 3 Results

### 3.1 Development and optimization of the AHQR–ddPCR assay

**Figure 1:**
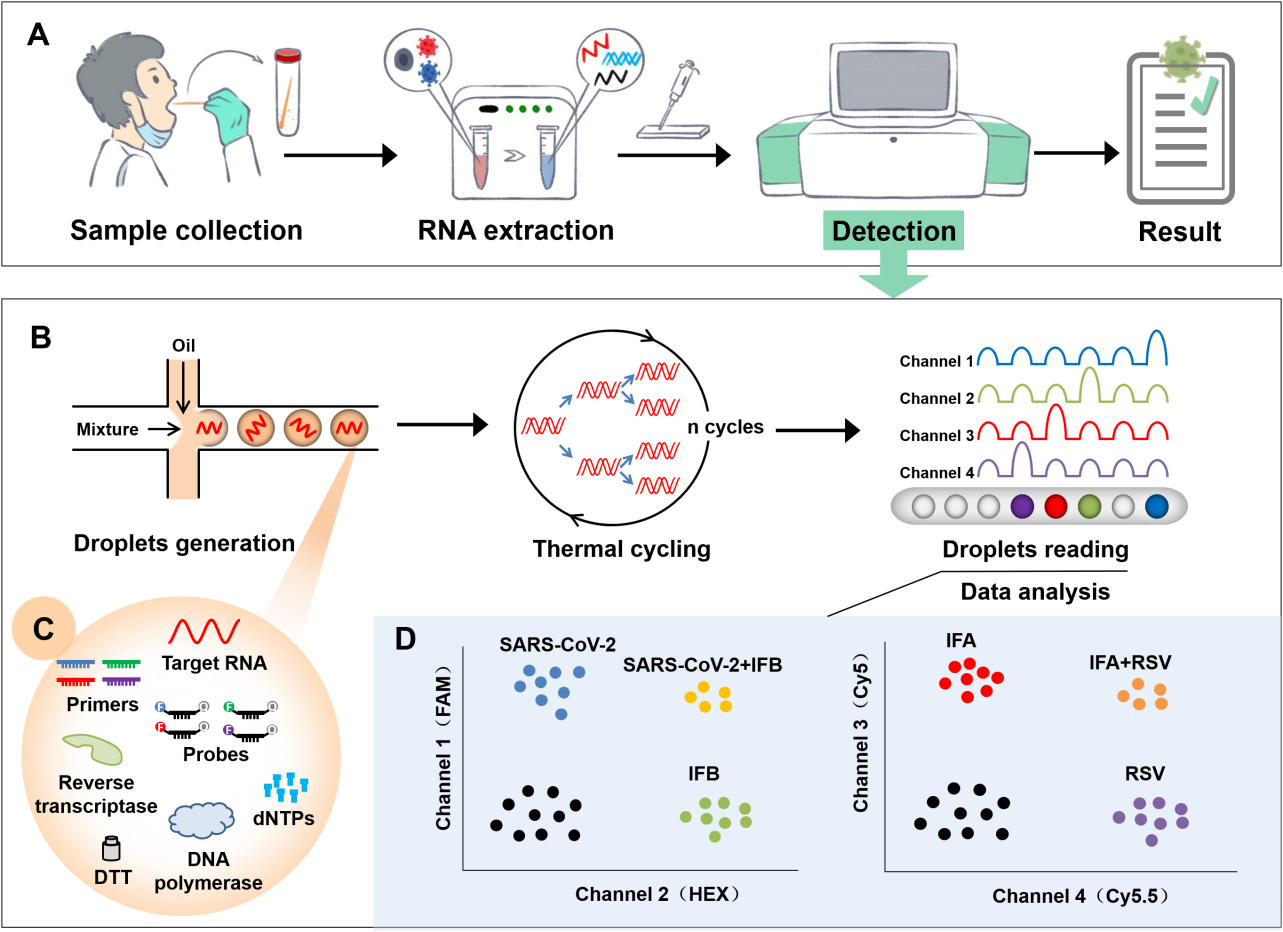
Flowchart of the newly established AHQR–ddPCR assay. **(A)** General workflow from sample collection to reporting result. **(B)** A standard RT–ddPCR workflow for detection, including droplets generation, thermal cycling, droplets reading, and data analysis. **(C)** Reaction mixture contained in each droplet. **(D)** Schematic representation of expected results when different targets were detected.

The developed AHQR-ddPCR assay can detect four respiratory viruses in one reaction, and the workflow from sample collection to reporting result was shown in **Figure 1A**. Samples from suspected infected patients were first collected and extracted before detection, followed by multiplex detection and absolute quantification with the hands-free QX ONE system. The standard RT–ddPCR workflow consisted of four stages: droplets generation, thermal cycling, droplets reading, and data analysis (**Figure 1B**). Starting with droplets generation, the prepared reaction mixture was divided into tens of thousands of droplets, each of which contained a separate reaction mixture to ensure that thousands of reactions occurred simultaneously (**Figure 1C**). The system then automatically performed end-point thermal cycling and measured the fluorescence amplitude of each droplet after amplification, and set a threshold to separate the droplets as either negative or positive (**Figure 1D**). Finally, the results of the analysis are calculated by the system software using Poisson statistics and expressed as copies per microliter of the reaction mixture.

The reaction conditions were optimized to maximize the performance of the assay. We first compared differences in the copies and droplet separation by setting different primer and probe concentrations as the different combinations directly affect the fluorescence amplitude (**Supplementary Figure 1**). The optimal concentrations of the primers and probe for IFA, IFB, RSV, and SARS-CoV-2 were 600 nM and 100 nM, 600 nM and 200 nM, 600 nM and 200 nM, 600 nM and 100 nM, respectively. We then optimized the different reverse transcription temperature (40–50°C) and time (10–30 min), annealing temperature (*Ta*, 50–60°C) and extension time (20–40 s). Based on the copies and the repeatability of results, the optimal temperature and time for reverse transcription were 48°C and 20 min, for *Ta* and extension time were 55°C and 30 s (**Supplementary Figure 2**).

### 3.2 Analytical sensitivity and specificity

**Figure 2:**
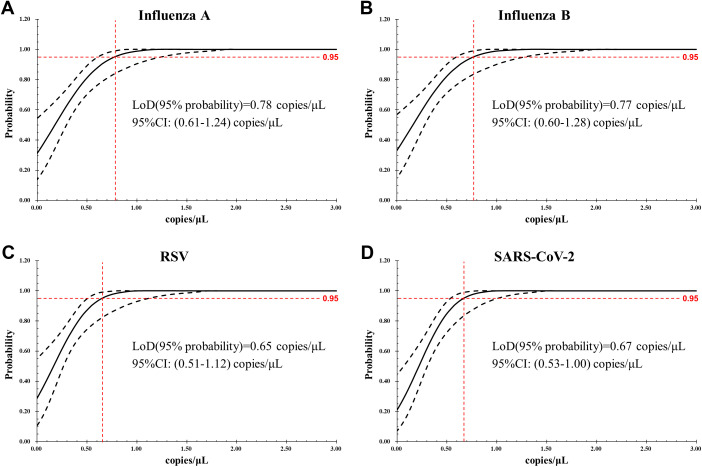
LoD of the AHQR–ddPCR assay was obtained by Probit analysis for four viruses: **(A)** Influenza A, **(B)** Influenza B, **(C)** RSV, **(D)** SARS-CoV-2. The X-axis shows expected concentration (copies/μL) and the Y-axis shows fraction of positive results in all parallel reactions performed. The inner line is a probit curve and the outer lines are 95% confidence interval (95% CI).

When LoB was evaluated by analyzing blank samples, the target genes of IFA, IFB, RSV, and SARS-CoV-2 were not detected in any of the 60 tests, giving LoB =0 copy/μL. The LoD for each target of the assay, determined by serial 2-fold dilutions of the reference materials, were as follows: 0.78 (95% confidence interval [CI]: 0.61–1.24) copies/μL for IFA, 0.77 (95% CI: 0.60–1.28) copies/μL for IFB, 0.65 (95% CI: 0.51–1.12) copies/μL for RSV, and 0.67 (95% CI: 0.53–1.00) copies/μL for SARS-CoV-2, respectively (**Figure 2**).

**Figure 3:**
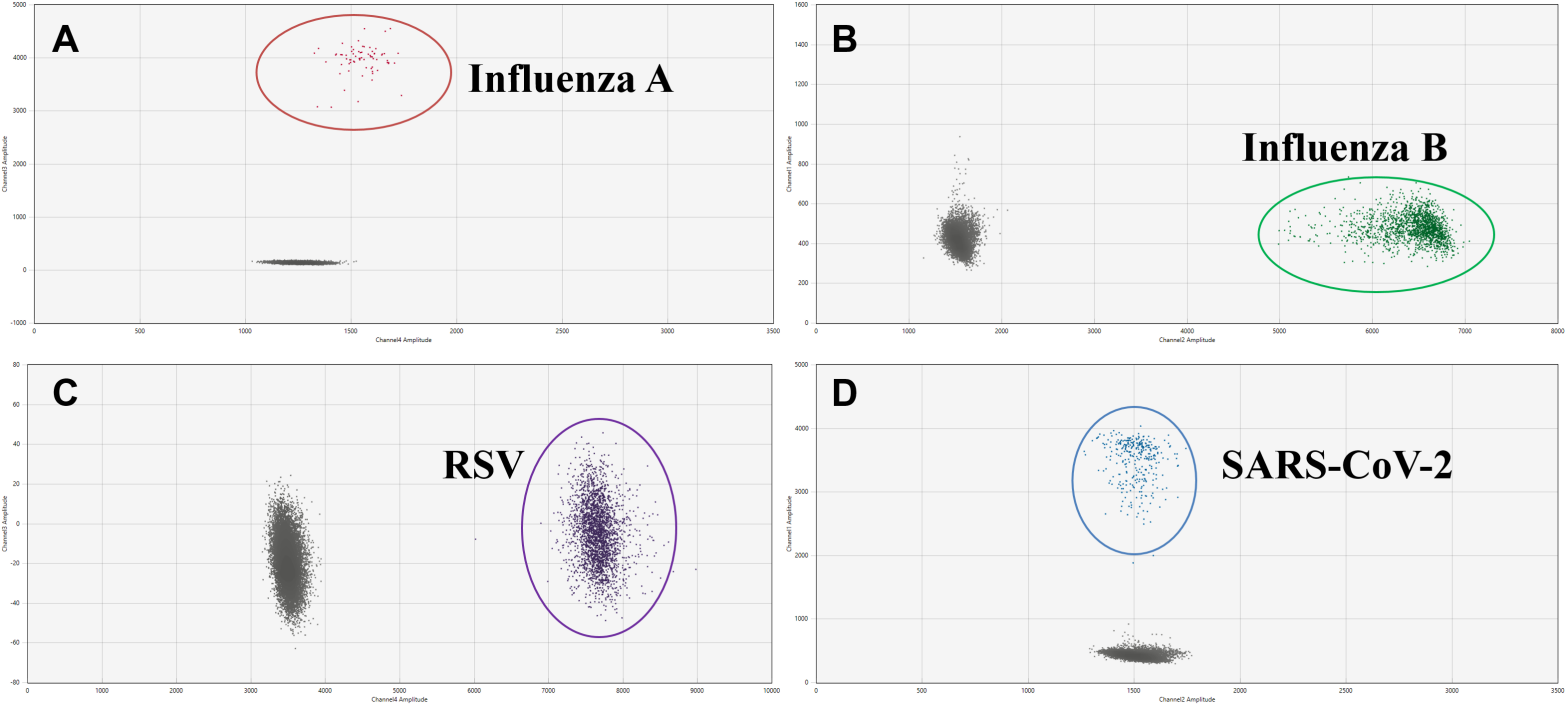
Two-dimensional (2D) plots of the analytical specificity of the AHQR–ddPCR assay for four viruses: **(A)** Influenza A, **(B)** Influenza B, **(C)** RSV, **(D)** SARS-CoV-2.

We next evaluated the analytical specificity of the assay to determine whether different pathogens could react with primer and probe sets. As shown in **Figure 3**, no cross-reactivity with the unintended targets was observed, indicating each pair of primers and probe was highly specific for the intended target.

### 3.3 Intraassay and interassay precision

The precision of the assay was determined by calculating the mean number of copies, SD, and CV% of two samples at different concentration (**Table 2**). For high-concentration sample (about 100 copies/μL), the CV% for the intraassay and interassay precision of four target genes ranged from 1.45% to 2.28% and 2.99% to 3.86%, respectively. For low-concentration sample (about 10 copies/μL), the CV% for the intraassay and interassay precision of four target genes ranged from 3.75% to 6.05% and 6.52% to 8.39%, respectively.

**Table 2:** Intraassay and interassay precision of the AHQR–ddPCR assay.

Target	Sample	Intraassay precision (n=20)		Interassay precision (n=50)	
		Mean ± SD (Copies/μL)	CV%	Mean ± SD (Copies/μL)	CV%
IFA	P1	107.29 ± 2.16	2.01	107.29 ± 3.21	2.99
	P2	14.18 ± 0.53	3.75	14.08 ± 0.92	6.52
IFB	P1	90.36 ± 1.31	1.45	91.10 ± 3.51	3.86
	P2	11.83 ± 0.51	4.35	11.88 ± 0.97	8.20
RSV	P1	88.34 ± 2.02	2.28	89.28 ± 2.99	3.34
	P2	12.37 ± 0.74	5.98	12.24 ± 1.03	8.39
SARS-CoV-2	P1	88.50 ± 1.56	1.77	88.33 ± 2.93	3.32
	P2	11.71 ± 0.71	6.05	11.89 ± 0.87	7.30

### 3.4 Linear dynamic range

**Figure 4:**
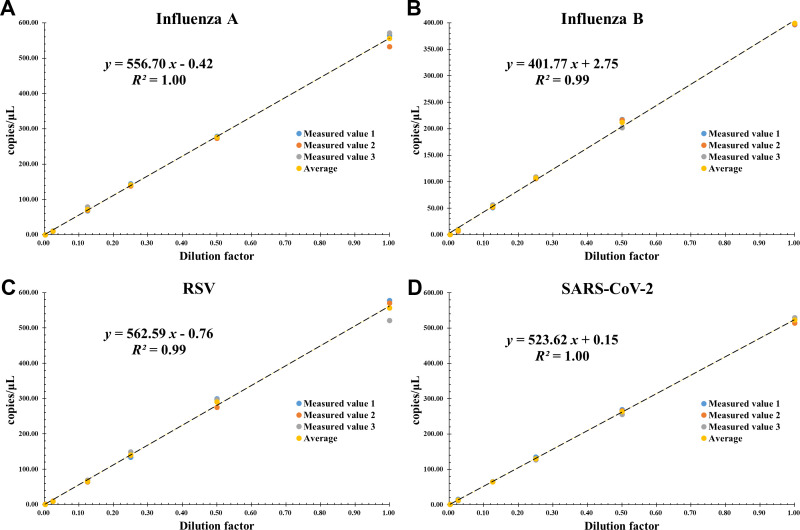
Linear dynamic range of the AHQR–ddPCR assay in serial 2-fold dilution from about 1000 to 1 copies/μL for four viruses: **(A)** Influenza A, **(B)** Influenza B, **(C)** RSV, **(D)** SARS-CoV-2. The X-axis represents the dilution factor and the Y-axis represents the absolute copies measured. The data points represent replicates of three independent experiments.

The linear regression plot between absolute copy number/μL (Y-axis) and dilution factor (X-axis) is shown in **Figure 4**. All reactions were performed in triplicate. For all target genes, the assay showed good linearity over the set concentration range of approximately 1.00-500 copies/μL. The slope of each curve ranged from 401.77 to 562.59, the intercept from -0.76 to +2.75, and the correlation coefficients (R^2^) were all ≥ 0.99, ensuring that the amplification efficiency of each target gene remained constant over a wide range of concentrations.

### 3.5 Performance on clinical samples and comparison with RT–PCR

A total of 247 clinical throat swab samples were analyzed simultaneously with both the developed assay and RT–PCR kits (**Supplementary Table 1**). The newly established AHQR–ddPCR assay demonstrated the sensitivity of the four targets compared to the actual results were 98.57% (95% CI: 92.30-99.96%) for IFA, 100.00% (95% CI: 93.84-100.00%) for IFB, 100.00% (95% CI: 92.75-100.00%) for RSV, and 97.06% (95% CI: 84.67-99.93%) for SARS-CoV-2, respectively. With RT–PCR, the sensitivity of the four targets were 94.29% (95% CI: 86.01-98.42%) for IFA, 98.28% (95% CI: 90.76-99.96%) for IFB, 93.88% (95% CI: 83.13-98.72%) for RSV, and 97.06% (95% CI: 84.67-99.93%) for SARS-CoV-2, respectively. In addition, both two assays exhibited excellent specificity. The concordance between the RT–ddPCR and RT–PCR assays was 97.98% (242/247, *K* = 0.95) for IFA, 99.60% (246/247, *K* = 0.99) for IFB, 98.79% (244/247, *K*= 0.96) for RSV, and 99.19% (245/247, *K* = 0.97) for SARS-CoV-2 (**Table 3**).

**Table 3:** Clinical diagnostic performance of the AHQR–ddPCR and RT–PCR assay (n=247).

Target	Assay	No. of results by actual type (n=247)				Sensitivity (95% CI)	Specificity (95% CI)	Concordance
		TP	FP	TN	FN			
IFA	RT-ddPCR	69	0	177	1	98.57% (92.30-99.96%)	100.00% (97.94-100.00%)	97.98% (242/247), *K*=0.95, *P*<0.001
	RT-PCR	66	0	177	4	94.29% (86.01-98.42%)	100.00% (97.94-100.00%)	
IFB	RT-ddPCR	58	0	189	0	100.00% (93.84-100.00%)	100.00% (98.07-100.00%)	99.60% (246/247), *K*=0.99, *P*<0.001
	RT-PCR	57	0	189	1	98.28% (90.76-99.96%)	100.00% (98.07-100.00%)	
RSV	RT-ddPCR	49	0	198	0	100.00% (92.75-100.00%)	100.00% (98.15-100.00%)	98.79% (244/247), *K*=0.96, *P*<0.001
	RT-PCR	46	0	198	3	93.88% (83.13-98.72%)	100.00% (98.15-100.00%)	
SARS-CoV-2	RT-ddPCR	33	0	213	1	97.06% (84.67-99.93%)	100.00% (98.28-100.00%)	99.19% (245/247), *K*=0.97, *P*<0.001
	RT-PCR	33	0	213	1	97.06% (84.67-99.93%)	100.00% (98.28-100.00%)	

TP, True Positive; FP, False Positive; TN, True Negative; FN, False Negative.

Although the overall concordance between RT–ddPCR and RT–PCR was good, 4.45% (11/247) of the results were still inconsistent. In detail, nine samples with concentrations <3.00 copies/μL were positive by RT–ddPCR but negative by RT–PCR, including four for IFA, one for IFB, three for RSV, and one for SARS-CoV-2. Two samples were negative by RT–ddPCR but positive by RT–PCR, including one for IFA and one for SARS-CoV-2. We then retested these eleven samples with tNGS and found that all the samples were positive, including two SARS-CoV-2 Omicron B.1.1.529, four IFA H1N1pdm09, one IFA H3N2, one IFB Victoria, and three RSV B. Notably, co-infection was identified in 4.05% (10/247) of samples based on the results of both assays, seven of which were found by RT–ddPCR and proved by RT–PCR, and the other three samples were found by RT–PCR, specifically three samples with IFA and RSV, two samples with IFA and IFB, one sample with IFA and SARS-CoV-2, one sample with IFB and SARS-CoV-2, two samples with PIV and RSV, and one sample with PIV and SARS-CoV-2.

## 4 Discussion

The high infectivity and pathogenicity of respiratory viruses highlight the urgent need to develop a more sensitive, accurate and reliable assay for their detection. Although RT–PCR has proven to be the most effective methods for identifying pathogens, it still has some limitations in detecting weakly positive samples and resisting inhibitors (Buchan and Ledeboer, 2014; Wang et al., 2020), which can be overcome with RT–ddPCR demonstrated by previous studies (Cassinari et al., 2021; Pichon et al., 2017; Zhang et al., 2022). However, the widely used QX200 platform in these studies can only detect two targets in separate channels and cannot meet the need for automation and high-throughput. Therefore, we established an AHQR–ddPCR assay that provides excellent performance for absolute quantification of four most frequently respiratory viruses in one reaction, ultimately facilitating the early triage and treatment of infected patients.

The newly established and optimized AHQR–ddPCR assay has excellent sensitivity and specificity, and can accurately and absolutely quantify different targets over a relatively wide range of concentrations. In particular, this automated system with independent four-color fluorescence channels displays superior performance in terms of precision and LoD. Good repeatability is the first prerequisite for reliable clinical results and plays an important role in ensuring the quality of pathogen detection. Our results demonstrate that the CV% of all the target genes in low-concentration samples ranged from 3.75% to 8.39%, larger than the CV% of high-concentration samples but still less than the 10%-25% reported in most studies (Kinloch et al., 2021; Leong et al., 2023). In clinical practice, better sensitivity increase the rate of successful detection and reduces the number of false-negative results, ultimately contributing to the early diagnosis and treatment of infected individuals. The LoD for four target genes (0.65–0.78 copies/μL) were lower than those previously reports (Nyaruaba et al., 2021; Suo et al., 2020; Zafeiriadou et al., 2024), indicating that the assay is particularly suitable for detecting low-concentration samples. The linear dynamic range of this assay is narrow at about 1.00-500 copies/μL, which is a distinctive technical feature of ddPCR compared to PCR. Although there are strategies to increase the range (Quan et al., 2018), they can lead to a decrease in sensitivity, so a narrower linear dynamic range is acceptable considering that ddPCR is mainly used to detect low-concentrations samples.

To analyze the clinical applicability of the assay, we compared the RT–ddPCR results in parallel with those obtained by RT–PCR. The results indicate that the established AHQR–ddPCR assay has superior diagnostic sensitivity than RT–PCR for IFA, IFB, and RSV, especially in low-concentration samples, which can significantly reduce the false-negatives results. Unlike other studies (Cassinari et al., 2021; Suo et al., 2020), the discrepancy of positivity rate was not evident for SARS-CoV-2. We speculate the main reason is that this assay only detects the ORF1ab gene of SARS-CoV-2, but previous studies have shown that the diagnostic sensitivity of ORF1ab gene is slightly less than that of the N gene (Suo et al., 2020; Zhang et al., 2023). Therefore, the key to solve this problem is to develop a higher-order multiplex assay that can detect multiple targets for each virus. In addition, the occurrence of six co-infected samples in this study further highlights the clinical value of multiplex detection, because co-infection can directly affect the course of the disease leading to an increased risk of complications and death especially during the epidemics.

Until now, no study has reported a RT–ddPCR assay based on the automated and high-throughput system, which has the advantage of minimizing personal errors, maximizing resource savings and time of the analysis. The entire process of this assay takes about 6.5 hours for 96 samples from sample RNA extraction to final results, shorter than 8 hours of the QX200 system (Nyaruaba et al., 2022; Tan et al., 2023). In practice, up to 480 samples of 5-plate capacity can be detected simultaneously in just 20 hours, which significantly reduces sample turnaround time. Although the overall detection time for 96 samples is longer than 3 hours of RT–PCR, it is acceptable considering the special advantages of RT–ddPCR such as high sensitivity, high precision, and absolute quantification. Particularly, this assay has great potential to be used as a quantitative tool for monitoring treatment and virology researches, which can complement traditional methods and even eliminate discrepancies in results from different laboratories. It is worth noting that although the entire workflow is automated, thresholds and droplet counts are of interest and need to be confirmed manually by professional technicians.

In the clinical application of RT–ddPCR, it is recommended that samples be analyzed with RT–PCR, followed by further RT–ddPCR analysis of inconsistent or suspicious samples, to minimize costs or when higher accuracy, better sensitivity, and multiplexing are required, such as public health surveillance, complex infections diagnosis, and viral load analysis (Nyaruaba et al., 2022). This study also has its own limitations. The results obtained by evaluating clinical performance using relatively small amounts of sample are not comprehensive, especially in the absence of different sample types like saliva. Another shortcoming is that endogenous reference gene, like the common glyceraldehyde-3-phosphate dehydrogenase (GAPDH) (Sharan et al., 2015), were not designed considering the practical scene using RT–ddPCR described above. Next, we plan to use additional strategies based on probe mixing or amplitude-based multiplexing to develop higher-order multiplex assays using more samples in the future research.

In conclusion, we have established an automated and high-throughput quadruplex RT–ddPCR assay, which has the advantages of high precision, sensitivity, and specificity. More importantly, we have successfully used this assay to identify the pathogens in clinical samples. Our results confirm the excellent clinical performance of this assay. This newly developed assay will facilitate the diagnosis of respiratory infections with similar symptoms and suppression of viral transmission, and provide a reference for the development of automated molecular diagnostics.

## Data Availability

The datasets presented in this study can be found in online repositories. The names of the repository/repositories and accession number(s) can be found in the article/**Supplementary Material**.

## Supplementary material

Supplementary Figure 1 The optimization of different primer and probe concentrations for the AHQR-ddPCR assay.

Supplementary Figure 2 The optimization of different reverse transcription temperature (40-50°C) and time (10-30 min), annealing temperature (*Ta*, 50-60°C) and extension time (20-40 s).

Supplementary Table 1 The results of 247 clinical samples detected by the established RT-ddPCR assay and RT-PCR simultaneously.
