# Supplementary material for: Development and validation of an automated and high-throughput quadruplex RT–ddPCR assay for the detection of influenza A, influenza B, respiratory syncytial virus, and SARS-CoV-2
Source: Front Cell Infect Microbiol. 2025 Mar 28;15:1529336. doi: 10.3389/fcimb.2025.1529336 (PMC11985766; doi:10.3389/fcimb.2025.1529336)
Supplement: Supplementary Figure 1 — The optimization of different primer and probe concentrations for the AHQR-ddPCR assay. [file Table1.docx]

**Development and validation of an automated and high-throughput quadruplex RT‐ddPCR assay for the detection of Influenza A, Influenza B, Respiratory Syncytial Virus, and SARS-CoV-2**

**Supplementary materials**


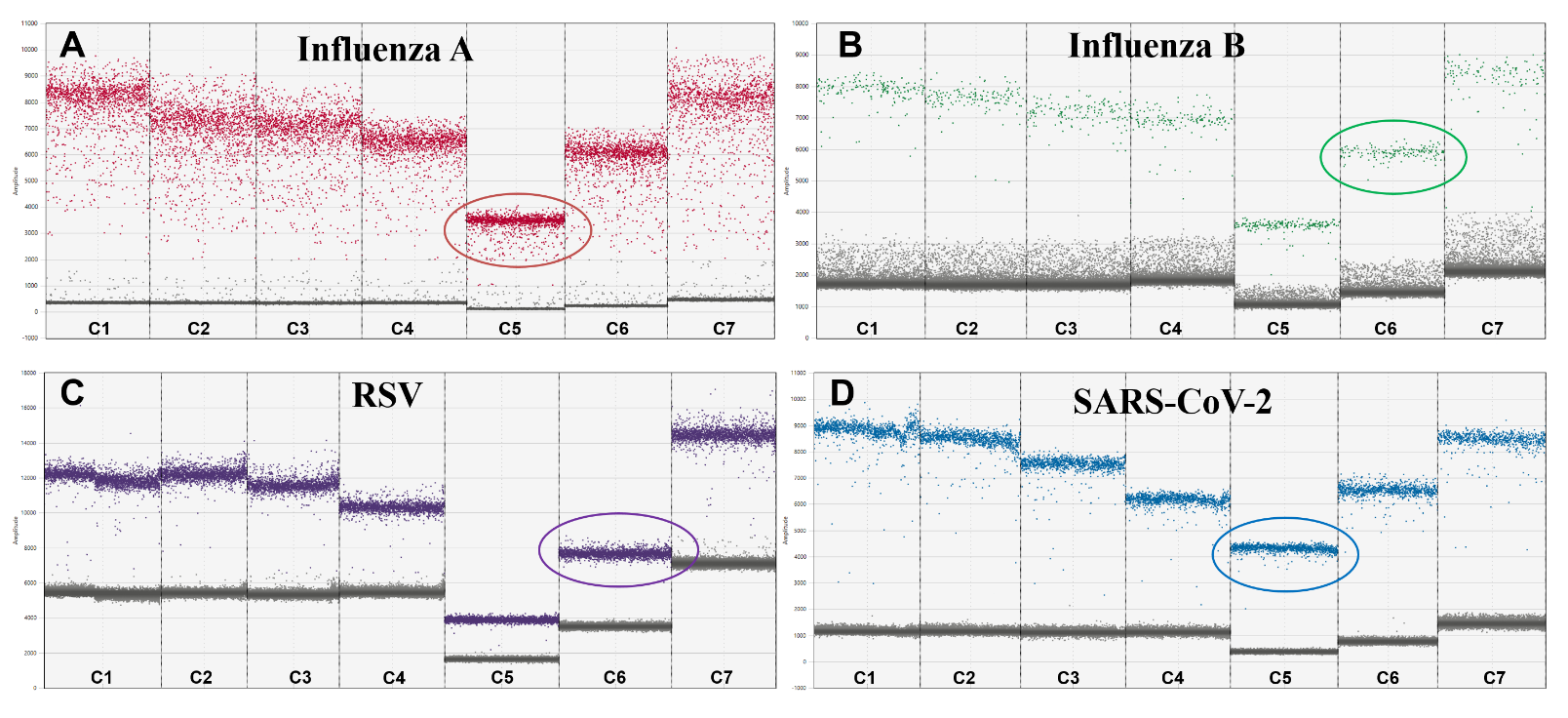


Supplementary Figure 1. The optimization of different primer and probe concentrations for the AHQR-ddPCR assay. The X-axis shows different primer-probe concentration combinations (C1-C7), and the Y-axis shows the distribution of droplets. Droplets plots of 7 different primer-probe concentration combinations (1000 nM and 300 nM for C1, 800 nM and 300 nM for C2, 600 nM and 300 nM for C3, 400 nM and 300 nM for C4, 600 nM and 100 nM for C5, 600 nM and 200 nM forC6, 600 nM and 400 nM for C7) for four viruses: (A) Influenza A, (B) Influenza B, (C) RSV, (D) SARS-CoV-2. Abbreviations: nM, nmol/L.


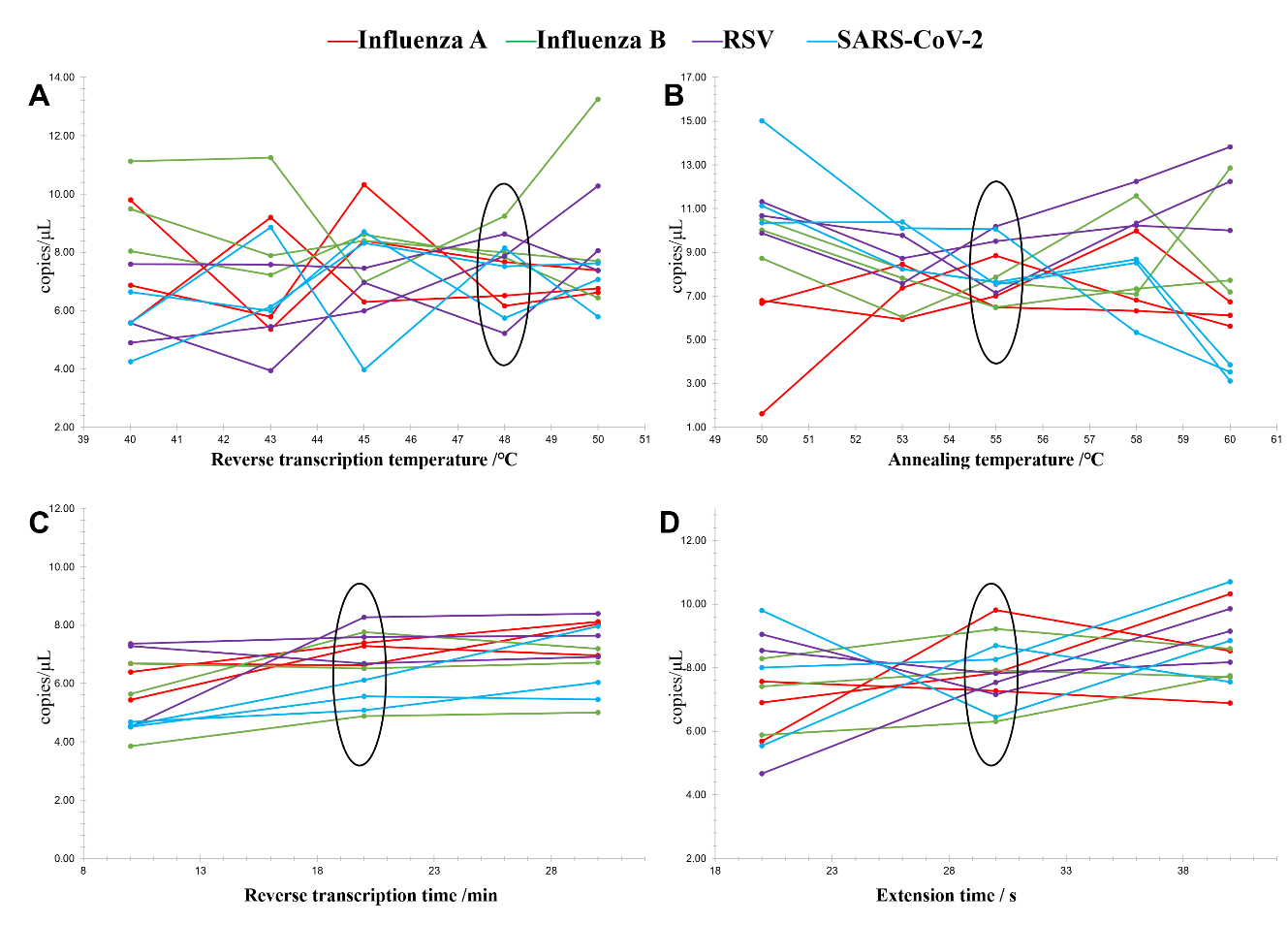


Supplementary Figure 2. The optimization of different reverse transcription temperature (40-50 ℃) and time (10-30 min), annealing temperature (*Ta*, 50-60 ℃) and extension time (20-40 s). Assay results under different reaction conditions: (A) Reverse transcription temperature, (B) Annealing temperature, (C) Reverse transcription time, (D) Extension time.

Supplementary Table 1. The results of 247 clinical samples detected by the established AHQR-ddPCR assay and RT-PCR simultaneously. Yellow shading represents inconsistent results between the two methods, and blue shading represents co-infections. Abbreviations: N, Not detected.

| No. | RT-ddPCR (concentrations, copies/μL) | | | | RT-PCR (Cycle threshold value, Ct) | | | | | | | | |
| --- | --- | --- | --- | --- | --- | --- | --- | --- | --- | --- | --- | --- | --- |
|  | IFA | IFB | RSV | SARS-CoV-2 | IFA | IFB | RSV | SARS-CoV-2 | PIV | HMPV | ADV | RhV | MP |
| 1 | N | N | N | 1.20 | N | N | N | 34.37 | N | N | N | N | N |
| 2 | N | N | N | 30.42 | N | N | N | 29.35 | N | N | N | N | N |
| 3 | N | N | N | 6236.76 | N | N | N | 22.64 | N | N | N | N | N |
| 4 | N | N | N | 4810.39 | N | N | N | 23.10 | N | N | N | N | N |
| 5 | N | N | N | 0.87 | N | N | N | N | N | N | N | N | N |
| 6 | N | N | N | 1.15 | N | N | N | 33.30 | N | N | N | N | N |
| 7 | N | N | N | N | N | N | N | 35.15 | N | N | N | N | N |
| 8 | N | N | N | 12.98 | N | N | N | 30.90 | N | N | N | N | N |
| 9 | N | N | N | 8.93 | N | N | N | 31.59 | N | N | N | N | N |
| 10 | N | N | N | 71.28 | N | N | N | 29.52 | N | N | N | N | N |
| 11 | N | N | N | 1.42 | N | N | N | 33.53 | N | N | N | N | N |
| 12 | N | N | N | >1000000 | N | N | N | 19.73 | N | N | N | N | N |
| 13 | N | N | N | 3.44 | N | N | N | 33.20 | N | N | N | N | N |
| 14 | N | N | N | 9.41 | N | N | N | 31.64 | N | N | N | N | N |
| 15 | N | N | N | 1.46 | N | N | N | 33.80 | N | N | N | N | N |
| 16 | N | N | N | 14.71 | N | N | N | 31.01 | N | N | N | N | N |
| 17 | N | N | N | 125.26 | N | N | N | 27.23 | N | N | N | N | N |
| 18 | N | N | N | 1.43 | N | N | N | 34.22 | N | N | N | N | N |
| 19 | N | N | N | 71.44 | N | N | N | 28.64 | N | N | N | N | N |
| 20 | N | N | N | 53.15 | N | N | N | 28.88 | N | N | N | N | N |
| 21 | N | N | N | 2.03 | N | N | N | 34.24 | N | N | N | N | N |
| 22 | N | N | N | 2.68 | N | N | N | 34.01 | N | N | N | N | N |
| 23 | N | N | N | 3021.45 | N | N | N | 23.25 | N | N | N | N | N |
| 24 | N | N | N | 6.47 | N | N | N | 32.34 | N | N | N | N | N |
| 25 | N | N | N | 36.65 | N | N | N | 28.88 | N | N | N | N | N |
| 26 | N | N | N | 2.64 | N | N | N | 31.36 | N | N | N | N | N |
| 27 | N | N | N | 315.08 | N | N | N | 26.41 | N | N | N | N | N |
| 28 | N | 11794.04 | N | N | N | 20.46 | N | N | N | N | N | N | N |
| 29 | N | 12.79 | N | N | N | 32.79 | N | N | N | N | N | N | N |
| 30 | N | >1000000 | N | N | N | 20.76 | N | N | N | N | N | N | N |
| 31 | N | 11.73 | N | N | N | 32.85 | N | N | N | N | N | N | N |
| 32 | N | 12.24 | N | N | N | 33.29 | N | N | N | N | N | N | N |
| 33 | N | 19127.04 | N | N | N | 22.15 | N | N | N | N | N | N | N |
| 34 | N | 6423.90 | N | N | N | 23.78 | N | N | N | N | N | N | N |
| 35 | N | 28.19 | N | N | N | 31.69 | N | N | N | N | N | N | N |
| 36 | N | >1000000 | N | N | N | 19.29 | N | N | N | N | N | N | N |
| 37 | N | 12518.21 | N | N | N | 22.55 | N | N | N | N | N | N | N |
| 38 | N | 643.05 | N | N | N | 27.03 | N | N | N | N | N | N | N |
| 39 | N | 35.32 | N | N | N | 31.04 | N | N | N | N | N | N | N |
| 40 | N | 4.55 | N | N | N | 33.94 | N | N | N | N | N | N | N |
| 41 | N | 3237.40 | N | N | N | 24.69 | N | N | N | N | N | N | N |
| 42 | N | 46.09 | N | N | N | 31.12 | N | N | N | N | N | N | N |
| 43 | N | 20.21 | N | N | N | 32.08 | N | N | N | N | N | N | N |
| 44 | N | 1.82 | N | N | N | 33.03 | N | N | N | N | N | N | N |
| 45 | N | 10454.89 | N | N | N | 21.43 | N | N | N | N | N | N | N |
| 46 | N | 1.68 | N | N | N | 32.63 | N | N | N | N | N | N | N |
| 47 | N | 7.87 | N | N | N | 31.71 | N | N | N | N | N | N | N |
| 48 | N | 3116.17 | N | N | N | 23.19 | N | N | N | N | N | N | N |
| 49 | N | 18.43 | N | N | N | 30.27 | N | N | N | N | N | N | N |
| 50 | N | 13871.00 | N | N | N | 21.03 | N | N | N | N | N | N | N |
| 51 | N | 15.63 | N | N | N | 30.45 | N | N | N | N | N | N | N |
| 52 | N | 40680.73 | N | N | N | 18.42 | N | N | N | N | N | N | N |
| 53 | N | 9171.19 | N | N | N | 21.75 | N | N | N | N | N | N | N |
| 54 | N | 6.44 | N | N | N | 31.48 | N | N | N | N | N | N | N |
| 55 | N | 31.82 | N | N | N | 29.75 | N | N | N | N | N | N | N |
| 56 | N | 20.36 | N | N | N | 30.32 | N | N | N | N | N | N | N |
| 57 | N | 589.69 | N | N | N | 25.89 | N | N | N | N | N | N | N |
| 58 | N | 27644.90 | N | N | N | 20.02 | N | N | N | N | N | N | N |
| 59 | N | N | N | N | 37.56 | N | N | N | N | N | N | N | N |
| 60 | 1.56 | N | N | N | 33.93 | N | N | N | N | N | N | N | N |
| 61 | 5.19 | N | N | N | 32.11 | N | N | N | N | N | N | N | N |
| 62 | 1.32 | N | N | N | N | N | N | N | N | N | N | N | N |
| 63 | 72.19 | N | N | N | 27.33 | N | N | N | N | N | N | N | N |
| 64 | 9.66 | N | N | N | 31.36 | N | N | N | N | N | N | N | N |
| 65 | 1.27 | N | N | N | N | N | N | N | N | N | N | N | N |
| 66 | 3.78 | N | N | N | 33.33 | N | N | N | N | N | N | N | N |
| 67 | 38.37 | N | N | N | 28.68 | N | N | N | N | N | N | N | N |
| 68 | 27.73 | N | N | N | 29.57 | N | N | N | N | N | N | N | N |
| 69 | 1.03 | N | N | N | 35.36 | N | N | N | N | N | N | N | N |
| 70 | 9558.85 | N | N | N | 20.36 | N | N | N | N | N | N | N | N |
| 71 | 279.90 | N | N | N | 25.09 | N | N | N | N | N | N | N | N |
| 72 | 15.33 | N | N | 98.19 | 29.14 | N | N | 28.19 | N | N | N | N | N |
| 73 | 47668.42 | N | N | N | 15.28 | N | N | N | N | N | N | N | N |
| 74 | 1.64 | N | N | N | 33.57 | N | N | N | N | N | N | N | N |
| 75 | 6.02 | N | N | N | 30.30 | N | N | N | N | N | N | N | N |
| 76 | 3.35 | N | N | N | 31.21 | N | N | N | N | N | N | N | N |
| 77 | 3.46 | N | N | N | 31.50 | N | N | N | N | N | N | N | N |
| 78 | 19.67 | N | N | N | 30.11 | N | N | N | N | N | N | N | N |
| 79 | 1.66 | N | N | N | 33.51 | N | N | N | N | N | N | N | N |
| 80 | 1.97 | N | N | N | 31.80 | N | N | N | N | N | N | N | N |
| 81 | 1.31 | N | N | N | 31.08 | N | N | N | N | N | N | N | N |
| 82 | 1.66 | N | N | N | 32.20 | N | N | N | N | N | N | N | N |
| 83 | 1.32 | N | N | N | 34.26 | N | N | N | N | N | N | N | N |
| 84 | 850.83 | N | N | N | 23.29 | N | N | N | N | N | N | N | N |
| 85 | 1.24 | N | N | N | 34.08 | N | N | N | N | N | N | N | N |
| 86 | 761.36 | N | N | N | 23.72 | N | N | N | N | N | N | N | N |
| 87 | 16.61 | N | N | N | 29.77 | N | N | N | N | N | N | N | N |
| 88 | 194.85 | N | 143.84 | N | 24.60 | N | 27.47 | N | N | N | N | N | N |
| 89 | 1.68 | N | N | N | 33.45 | N | N | N | N | N | N | N | N |
| 90 | 1.25 | N | 104.06 | N | 34.72 | N | 28.06 | N | N | N | N | N | N |
| 91 | N | N | 6807.97 | N | N | N | 23.95 | N | N | N | N | N | N |
| 92 | N | N | 6.36 | N | N | N | 33.94 | N | N | N | N | N | N |
| 93 | N | N | 8383.93 | N | N | N | 23.51 | N | N | N | N | N | N |
| 94 | N | N | 8.26 | N | N | N | 35.53 | N | N | N | N | N | N |
| 95 | N | N | 2.50 | N | N | N | N | N | N | N | N | N | N |
| 96 | N | N | 2.88 | N | N | N | 35.43 | N | N | N | N | N | N |
| 97 | N | N | 5090.90 | N | N | N | 23.57 | N | N | N | N | N | N |
| 98 | N | N | 7.00 | N | N | N | 33.42 | N | N | N | N | N | N |
| 99 | N | N | 0.92 | N | N | N | 38.62 | N | N | N | N | N | N |
| 100 | N | N | 3.48 | N | N | N | 35.44 | N | N | N | N | N | N |
| 101 | N | N | 126.93 | N | N | N | 30.63 | N | N | N | N | N | N |
| 102 | N | N | 2656.09 | N | N | N | 25.21 | N | N | N | N | N | N |
| 103 | N | N | 10850.37 | N | N | N | 25.54 | N | N | N | N | N | N |
| 104 | N | N | 2.64 | N | N | N | 35.27 | N | N | N | N | N | N |
| 105 | N | N | 830.34 | N | N | N | 25.33 | N | N | N | N | N | N |
| 106 | N | N | 7756.78 | N | N | N | 23.06 | N | N | N | N | N | N |
| 107 | N | N | 6.41 | N | N | N | 32.93 | N | N | N | N | N | N |
| 108 | N | N | 117.58 | N | N | N | 29.03 | N | N | N | N | N | N |
| 109 | N | N | 355.44 | N | N | N | 26.52 | N | N | N | N | N | N |
| 110 | N | N | 1.02 | N | N | N | N | N | N | N | N | N | N |
| 111 | N | N | 16.93 | N | N | N | 32.87 | N | N | N | N | N | N |
| 112 | N | N | 0.97 | N | N | N | 37.14 | N | N | N | N | N | N |
| 113 | N | N | 1.29 | N | N | N | 36.47 | N | N | N | N | N | N |
| 114 | N | N | 3732.15 | N | N | N | 23.15 | N | N | N | N | N | N |
| 115 | N | N | 694.86 | N | N | N | 27.50 | N | N | N | N | N | N |
| 116 | N | N | 5.49 | N | N | N | 32.57 | N | N | N | N | N | N |
| 117 | N | N | 36.58 | N | N | N | 30.69 | N | N | N | N | N | N |
| 118 | N | N | 146.83 | N | N | N | 29.00 | N | N | N | N | N | N |
| 119 | N | N | 227.49 | N | N | N | 27.88 | N | N | N | N | N | N |
| 120 | N | N | 2.89 | N | N | N | N | N | N | N | N | N | N |
| 121 | N | N | 5.11 | N | N | N | 34.34 | N | N | N | N | N | N |
| 122 | N | N | 2683.37 | N | N | N | 25.20 | N | N | N | N | N | N |
| 123 | N | N | 136.99 | N | N | N | 29.14 | N | N | N | N | N | N |
| 124 | N | N | 1.96 | N | N | N | 37.25 | N | N | N | N | N | N |
| 125 | N | N | 2.75 | N | N | N | 35.02 | N | N | N | N | N | N |
| 126 | N | N | N | 43.38 | N | N | N | 29.71 | 35.09 | N | N | N | N |
| 127 | N | N | N | N | N | N | N | N | 25.25 | N | N | N | N |
| 128 | N | N | N | N | N | N | N | N | 25.64 | N | N | N | N |
| 129 | N | N | N | N | N | N | N | N | 26.69 | N | N | N | N |
| 130 | N | N | N | N | N | N | N | N | 25.21 | N | N | N | N |
| 131 | N | N | 1.07 | N | N | N | 38.25 | N | 30.95 | N | N | N | N |
| 132 | N | N | N | N | N | N | N | N | 38.09 | N | N | N | N |
| 133 | N | N | N | N | N | N | N | N | 20.94 | N | N | N | N |
| 134 | N | N | N | N | N | N | N | N | 26.09 | N | N | N | N |
| 135 | N | N | N | N | N | N | N | N | 26.76 | N | N | N | N |
| 136 | N | N | 8.03 | N | N | N | 33.89 | N | 37.45 | N | N | N | N |
| 137 | N | N | N | N | N | N | N | N | 27.71 | N | N | N | N |
| 138 | N | N | N | N | N | N | N | N | 32.16 | N | N | N | N |
| 139 | N | N | N | N | N | N | N | N | N | 37.96 | N | N | N |
| 140 | N | N | N | N | N | N | N | N | N | 30.24 | N | N | N |
| 141 | N | N | N | N | N | N | N | N | N | 28.76 | N | N | N |
| 142 | N | N | N | N | N | N | N | N | N | 29.58 | N | N | N |
| 143 | N | N | N | N | N | N | N | N | N | N | 23.58 | N | N |
| 144 | N | N | N | N | N | N | N | N | N | N | 19.77 | N | N |
| 145 | N | N | N | N | N | N | N | N | N | N | 28.65 | N | N |
| 146 | N | N | N | N | N | N | N | N | N | N | 22.63 | N | N |
| 147 | N | N | N | N | N | N | N | N | N | N | 26.38 | N | N |
| 148 | N | N | N | N | N | N | N | N | N | N | 20.48 | N | N |
| 149 | N | N | N | N | N | N | N | N | N | N | 25.96 | N | N |
| 150 | N | N | N | N | N | N | N | N | N | N | N | 27.92 | N |
| 151 | N | N | N | N | N | N | N | N | N | N | N | 31.65 | N |
| 152 | N | N | N | N | N | N | N | N | N | N | N | 29.22 | N |
| 153 | N | N | N | N | N | N | N | N | N | N | N | N | 38.92 |
| 154 | N | N | N | N | N | N | N | N | N | N | N | N | 37.78 |
| 155 | N | N | N | N | N | N | N | N | N | N | N | N | 35.60 |
| 156 | N | N | N | N | N | N | N | N | N | N | N | N | 35.31 |
| 157 | N | N | N | N | N | N | N | N | N | N | N | N | 36.42 |
| 158 | N | N | N | N | N | N | N | N | N | N | N | N | 35.36 |
| 159 | N | N | N | N | N | N | N | N | N | N | N | N | 36.28 |
| 160 | N | N | N | N | N | N | N | N | N | N | N | N | 35.87 |
| 161 | N | N | N | N | N | N | N | N | N | N | N | N | 38.94 |
| 162 | N | N | N | N | N | N | N | N | N | N | N | N | 27.62 |
| 163 | N | N | N | N | N | N | N | N | N | N | N | N | 27.57 |
| 164 | N | N | N | N | N | N | N | N | N | N | N | N | 38.34 |
| 165 | N | N | N | N | N | N | N | N | N | N | N | N | 30.09 |
| 166 | N | N | N | N | N | N | N | N | N | N | N | N | 30.64 |
| 167 | N | N | N | N | N | N | N | N | N | N | N | N | 35.42 |
| 168 | N | N | N | N | N | N | N | N | N | N | N | N | 35.55 |
| 169 | N | N | N | N | N | N | N | N | N | N | N | N | 37.11 |
| 170 | N | N | N | N | N | N | N | N | N | N | N | N | 36.09 |
| 171 | N | N | N | N | N | N | N | N | N | N | N | N | 34.87 |
| 172 | N | N | N | N | N | N | N | N | N | N | N | N | 35.65 |
| 173 | 524.25 | N | N | N | 29.12 | N | N | N | N | N | N | N | N |
| 174 | 11.09 | N | N | N | 34.11 | N | N | N | N | N | N | N | N |
| 175 | 5.16 | N | N | N | 35.37 | N | N | N | N | N | N | N | N |
| 176 | 1.23 | N | N | N | 37.44 | N | N | N | N | N | N | N | N |
| 177 | 40.70 | N | N | N | 32.09 | N | N | N | N | N | N | N | N |
| 178 | 10.40 | N | N | N | 34.56 | N | N | N | N | N | N | N | N |
| 179 | 2.61 | N | N | N | 37.88 | N | N | N | N | N | N | N | N |
| 180 | 0.96 | N | N | N | 38.77 | N | N | N | N | N | N | N | N |
| 181 | 2249.03 | N | N | N | 25.95 | N | N | N | N | N | N | N | N |
| 182 | 9.19 | N | N | N | 34.48 | N | N | N | N | N | N | N | N |
| 183 | 208.52 | N | N | N | 30.11 | N | N | N | N | N | N | N | N |
| 184 | 1011.16 | N | N | N | 27.95 | N | N | N | N | N | N | N | N |
| 185 | 108.89 | N | N | N | 31.83 | N | N | N | N | N | N | N | N |
| 186 | 348.52 | 3.74 | N | N | 29.34 | 33.93 | N | N | N | N | N | N | N |
| 187 | 18.77 | N | N | N | 34.47 | N | N | N | N | N | N | N | N |
| 188 | 128.61 | N | N | N | 30.68 | N | N | N | N | N | N | N | N |
| 189 | 1.63 | N | N | N | 36.76 | N | N | N | N | N | N | N | N |
| 190 | 60.91 | N | N | N | 32.10 | N | N | N | N | N | N | N | N |
| 191 | 89.07 | N | N | N | 32.47 | N | N | N | N | N | N | N | N |
| 192 | 3.34 | N | N | N | 36.14 | N | N | N | N | N | N | N | N |
| 193 | 113.31 | N | N | N | 30.96 | N | N | N | N | N | N | N | N |
| 194 | 23.39 | N | N | N | 33.46 | N | N | N | N | N | N | N | N |
| 195 | 1.61 | N | N | N | 38.67 | N | N | N | N | N | N | N | N |
| 196 | 2328.58 | N | N | N | 26.45 | N | N | N | N | N | N | N | N |
| 197 | 1.88 | N | N | N | 36.89 | N | N | N | N | N | N | N | N |
| 198 | 1.59 | N | N | N | N | N | N | N | N | N | N | N | N |
| 199 | 1.07 | N | N | N | 37.94 | N | N | N | N | N | N | N | N |
| 200 | 5.27 | N | N | N | 36.30 | N | N | N | N | N | N | N | N |
|  |  |  |  |  |  |  |  |  |  |  |  |  |  |
| 201 | 12.85 | N | N | N | 34.25 | N | N | N | N | N | N | N | N |
| 202 | 3.32 | N | N | N | 37.05 | N | N | N | N | N | N | N | N |
| 203 | 3.97 | N | N | N | 35.19 | N | N | N | N | N | N | N | N |
| 204 | 1.03 | N | N | N | 38.25 | N | N | N | N | N | N | N | N |
| 205 | 1.26 | N | N | N | N | N | N | N | N | N | N | N | N |
| 206 | 22805.62 | N | N | N | 23.21 | N | N | N | N | N | N | N | N |
| 207 | 67.59 | 16.16 | N | N | 31.75 | 34.19 | N | N | N | N | N | N | N |
| 208 | 10000.00 | N | N | N | 16.97 | N | N | N | N | N | N | N | N |
| 209 | N | 4.57 | N | N | N | 37.33 | N | N | N | N | N | N | N |
| 210 | N | 28.51 | N | N | N | 33.07 | N | N | N | N | N | N | N |
| 211 | N | 4.28 | N | N | N | 35.87 | N | N | N | N | N | N | N |
| 212 | N | 11.24 | N | N | N | 36.44 | N | N | N | N | N | N | N |
| 213 | N | 21.95 | N | N | N | 33.22 | N | N | N | N | N | N | N |
| 214 | N | 382.86 | N | N | N | 29.47 | N | N | N | N | N | N | N |
| 215 | N | 1.24 | N | N | N | 39.14 | N | N | N | N | N | N | N |
| 216 | N | 249.23 | N | N | N | 29.98 | N | N | N | N | N | N | N |
| 217 | N | 13.90 | N | N | N | 33.30 | N | N | N | N | N | N | N |
| 218 | N | 1.25 | N | N | N | N | N | N | N | N | N | N | N |
| 219 | N | 1.39 | N | N | N | 38.96 | N | N | N | N | N | N | N |
| 220 | N | 2.12 | N | N | N | 38.79 | N | N | N | N | N | N | N |
| 221 | N | 10000.00 | N | N | N | 20.98 | N | N | N | N | N | N | N |
| 222 | N | 1.26 | N | N | N | 38.66 | N | N | N | N | N | N | N |
| 223 | N | 635.27 | N | N | N | 30.33 | N | N | N | N | N | N | N |
| 224 | N | 74.42 | N | N | N | 31.77 | N | N | N | N | N | N | N |
| 225 | N | 70.87 | N | N | N | 32.08 | N | N | N | N | N | N | N |
| 226 | N | 109.94 | N | N | N | 31.17 | N | N | N | N | N | N | N |
| 227 | N | 57.47 | N | N | N | 32.31 | N | N | N | N | N | N | N |
| 228 | N | 821.83 | N | N | N | 28.31 | N | N | N | N | N | N | N |
| 229 | N | 2.85 | N | N | N | 38.86 | N | N | N | N | N | N | N |
| 230 | N | 22.94 | N | N | N | 34.89 | N | N | N | N | N | N | N |
| 231 | N | 27.31 | N | N | N | 33.82 | N | N | N | N | N | N | N |
| 232 | N | 610.86 | N | 60.27 | N | 28.76 | N | 30.45 | N | N | N | N | N |
| 233 | N | 0.97 | N | N | N | 39.42 | N | N | N | N | N | N | N |
| 234 | N | N | 11.76 | N | N | N | 34.85 | N | N | N | N | N | N |
| 235 | N | N | 130.58 | N | N | N | 30.89 | N | N | N | N | N | N |
| 236 | N | N | 1.54 | N | N | N | 38.12 | N | N | N | N | N | N |
| 237 | 8.20 | N | 16.72 | N | 33.97 | N | 33.96 | N | N | N | N | N | N |
| 238 | N | N | 3.08 | N | N | N | 37.13 | N | N | N | N | N | N |
| 239 | N | N | 24.66 | N | N | N | 33.55 | N | N | N | N | N | N |
| 240 | N | N | 4.95 | N | N | N | 34.78 | N | N | N | N | N | N |
| 241 | N | N | 51.69 | N | N | N | 31.91 | N | N | N | N | N | N |
| 242 | N | N | 184.05 | N | N | N | 30.57 | N | N | N | N | N | N |
| 243 | N | N | 164.21 | N | N | N | 30.59 | N | N | N | N | N | N |
| 244 | N | N | N | 2.06 | N | N | N | 38.77 | N | N | N | N | N |
| 245 | N | N | N | 9201.27 | N | N | N | 25.43 | N | N | N | N | N |
| 246 | N | N | N | 62.66 | N | N | N | 29.77 | N | N | N | N | N |
| 247 | N | N | N | 49.40 | N | N | N | 30.77 | N | N | N | N | N |
